# Best Practices for Managing Patients with Unresectable Metastatic Gastric and Gastroesophageal Junction Cancer in Canada

**DOI:** 10.3390/curroncol31050191

**Published:** 2024-04-30

**Authors:** Stephanie Snow, Denise Gabrielson, Howard Lim, Mustapha Tehfe, Christine Brezden-Masley

**Affiliations:** 1 Division of Medical Oncology, Department of Medicine, Dalhousie University, QEII-Bethune Building, Suite 449 Bethune Building, 1276 South Park Street, Halifax, NS B3H 2Y9, Canada; 2 Division of Hematology/Oncology, St. Michael’s Hospital, 30 Bond Street, Toronto, ON M5B 1W8, Canada; 3 Division of Medical Oncology, BC Cancer Vancouver, 600 West 10th Avenue, Vancouver, BC V5Z 4E6, Canada; 4 Centre Hospitalier de l’Université de Montréal, 1000 Saint-Denis St, Montréal, QC H2X 0C1, Canada; moustapha.tehfe.med@ssss.gouv.qc.ca; 5 School of Medicine University of Toronto, Mount Sinai Hospital, 1284-600, University Avenue, Toronto, ON M5G 1X5, Canada; christine.brezden@sinaihealth.ca

**Keywords:** gastric cancer (GC), gastroesophageal junction cancer (GEJC), patient management, malnutrition, multidisciplinary care

## Abstract

Gastric cancer (GC) is one of the most common types of cancer and is associated with relatively low survival rates. Despite its considerable burden, there is limited guidance for Canadian clinicians on the management of unresectable metastatic GC and gastroesophageal junction cancer (GEJC). Therefore, we aimed to discuss best practices and provide expert recommendations for patient management within the current Canadian unresectable GC and GEJC landscape. A multidisciplinary group of Canadian healthcare practitioners was assembled to develop expert recommendations via a working group. The often-rapid progression of unresectable GC and GEJC and the associated malnutrition have a significant impact on the patient’s quality of life and ability to tolerate treatment. Hence, recommendations include early diagnosis, identification of relevant biomarkers to improve personalized treatment, and relevant support to manage comorbidities. A multidisciplinary approach including early access to registered dietitians, personal support networks, and palliative care services, is needed to optimize possible outcomes for patients. Where possible, patients with unresectable GC and GEJC would benefit from access to clinical trials and innovative treatments.

## 1. Introduction

Gastric cancer (GC) is the fifth most common cancer and the fourth leading cause of cancer-related deaths worldwide [1]. In Canada, there were approximately 4100 new cases in 2023, with an incidence rate of 8.6 per 100,000 people [2]. There are several risk factors for developing GC, the main risk being *Helicobacter pylori* infection [2]. Other risk factors include being male, an age of >50 years, and low socioeconomic status [2]. GC is most prevalent in Eastern Asia, where over 60% of global cases of gastric cancers are found. In Japan, GC ranks second in cancer incidence and third in mortality. However, population-based screening via endoscopy or radiography is conducted to facilitate early diagnosis, resulting in reduced mortality rates [3]. Screening in Japan starts at age 40, resulting in 53% of GCs being localized when diagnosed, as opposed to 27% of those in the United States [4]. In Canada, there is no screening program for GC, and diagnostic testing is typically initiated after the patient is symptomatic. Although survival rates have increased in high-income countries, GC survival remains relatively low, with a 5-year survival rate of 21–33% (in Canada, 29%) [2], and reports have documented increasing incidence in younger populations (<50 years old) [5,6,7].

GC encompasses two main topographical sites of origin in the stomach, including the proximal cardia (adjoining gastroesophageal junction [GEJ]) and the distal non-cardia stomach regions [8]. While these anatomical subsites have different etiologies, they share risk factors and are treated in similar ways in the advanced incurable setting [9]. The most common type of GC is adenocarcinoma, accounting for 95% of cases, with the remaining 5% of cases being considered rare tumor types (gastrointestinal stromal tumors [GISTs], neuroendocrine tumors [NETs], lymphomas, and adenosquamous carcinoma) [2]. In Canada, diagnosis of GC includes a physical examination, blood work, upper gastrointestinal endoscopy, biopsy, and tumor marker tests to assess relevant biomarkers needed to guide treatment, as well as a computerized tomography (CT) scan of the chest, abdomen, and pelvis and a chest X-ray [2].

The most common presenting symptoms are non-specific and include weight loss, persistent abdominal pain, dysphagia, hematemesis, anorexia, nausea, early satiety, and dyspepsia [10]. As a result, malnutrition is prevalent amongst patients with GC, with an estimated 48–80% of patients experiencing gastrointestinal cancer-related weight loss at diagnosis [11]. Malnutrition in this population is multifactorial. The presence of the primary tumor, metabolic abnormalities, or partial or complete gastrectomy can lead to loss of appetite, reduced food intake, poor digestion, dysmotility, and malabsorption [12]. Moreover, anorexia, reflux, bloating, nausea, and vomiting are common symptoms leading to weight loss and anemia [13]. Malnutrition has a significant impact on patients’ treatment tolerance, functioning, and quality of life (QoL) [14,15,16,17,18]. It has been independently associated with poor survival following gastric surgery, particularly among older populations [19,20]. Therefore, interventions to address malnutrition and other related symptoms, including psychosocial and palliative care, may benefit survival outcomes.

There is a paucity of guidance for the holistic management of unresectable metastatic GC and GEJ cancer (GEJC) in Canada. The objective of this report is, therefore, to discuss best practices and provide expert recommendations for patient management within the current Canadian unresectable metastatic GC and GEJC landscape.

## 2. Methods

A multidisciplinary group of Canadian healthcare practitioners, including four medical oncologists and one registered dietitian (RD) who are leaders in the field of GC, was assembled to develop expert recommendations via a working group. The authors identified current gaps in care and made recommendations to address these gaps.

## 3. Recommendations

The recommendations in Table 1 provide guidance to navigate personalized treatment and highlight the importance of multidisciplinary support from RDs and palliative care specialists, as well as personal support networks. These recommendations are based on the best available scientific evidence, clinical information, and professional expert opinions.

## 4. Impact of Comorbidities on Treatment

Recommendation 1. Physicians should determine the severity of comorbidities and their impacts on functional and performance status, and they should consider comorbidity management strategies prior to initiating treatment. The comorbidities associated with unresectable GC and GEJC, such as anemia, cardiovascular disease, and diabetes, in addition to age, are among the most important factors to consider when determining treatment options. Notably, there is a meaningful bi-modal age distribution in the presentation of unresectable GC and GEJC with a large group of patients, primarily female, presenting at 20–40 years of age and another presenting with advanced age (i.e., ≥70 years of age) [21]. Patients with advanced age can be challenging to treat due to possible frailty and poor functional status. However, while 70 years old is considered advanced age in clinical trials, disease management should not be based on age cut-offs, as there is no accepted definition for what describes an elderly patient when treating GC [22]. A patient’s functional status should be a primary consideration when determining treatment options. A poor Eastern Cooperative Oncology Group performance status (ECOG-PS) (e.g., ECOG ≥ 3) may indicate that a patient is unlikely to benefit from or tolerate aggressive treatments, including cytotoxic systemic therapy and surgery. If factors contributing to poor performance status, including malnutrition, iron deficiency anemia, suboptimal pain control, and disease-related nausea and vomiting, are addressed, it may improve the likelihood of successful interventions.

Pre-existing comorbidities can impact treatment choice and influence the use of chemotherapy. For instance, oxaliplatin or taxane chemotherapy can worsen pre-existing peripheral sensory neuropathy. Similarly, active or uncontrolled autoimmune comorbidities may make a patient a poor candidate for an immune checkpoint inhibitor. Finally, poor cardiac function is a contraindication to targeted therapy with trastuzumab in patients whose cancers overexpress the HER2/neu protein, and a referral to cardio-oncology is recommended to optimize cardiac function.

## 5. Diagnosis and Treatment-specific Considerations for Patient Management

### 5.1. Biomarker Testing

Recommendation 2. Reflexive biomarker testing, including mismatch repair proteins (MMRs), programmed death-ligand 1 (PD-L1)/combined positive score (CPS), and human epidermal growth factor receptor 2 (HER2), should be standard practice at all centers to improve personalized care and outcomes. Although zolbetuximab is not yet approved by Health Canada, Claudin 18.2 will also need to be reflex-tested, as this monoclonal antibody has demonstrated survival benefits when added to first-line chemotherapy for metastatic disease. Most cases of GC are diagnosed at advanced stages, increasing the likelihood of poor outcomes; therefore, testing biomarkers reflexively will allow for a more timely introduction of optimal targeted therapies to improve survival outcomes [23,24].

To date, there are four biomarkers that predict responses to targeted or immunotherapy treatment. The HER2+ biomarker is overexpressed in ~10–20% of metastatic GC and GEJC, microsatellite instability high (MSI-H) in approximately 11%, PD-L1 + CPS ≥ 5 using the Dako 28-8 assay in 60%, and CLDN18.2 in 38% of metastatic GC tumors [25,26,27,28]. Biomarker assessment is crucial for patient selection for optimal systemic therapy beyond chemotherapy. The cost burden associated with suboptimal treatment outweighs the cost of conducting comprehensive biomarker testing for all patients. While biomarker testing is recommended for all patients, the availability of biomarker testing is institution-specific and depends on provincial and private reimbursement coverage, access to validated testing, and human resource issues for testing and interpretation.

### 5.2. Treatment

**Figure 1:**
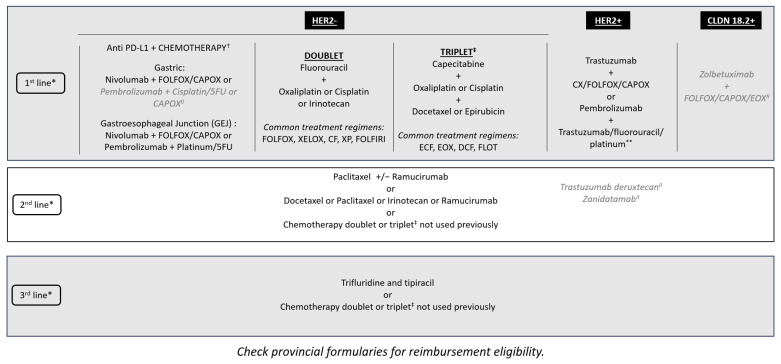
Treatment sequencing options for metastatic GC and GEJC within the Canadian landscape. * Clinical trials should be considered in all lines of therapy. ^✝^ Immunotherapy is funded regardless of PD-L1 CPS testing, but those who have a high PD-L1 CPS as defined by registrational clinical trials are most likely to benefit. ^‡^ Triplet therapy may be considered for younger, fit patients with normal organ function (ECOG: 0–1, <70 years old, minimal comorbidities) who are more able to tolerate increased toxicity, which may not reflect the patients in all practices. ^II^ Not approved by Health Canada for this indication. ** Limited to patients with a PD-L1 CPS of ≥ 1. CAPOX: capecitabine and oxaliplatin; CF: cisplatin and fluorouracil; CPS: combined positive score; CX: cisplatin and capecitabine; DCF: docetaxel, cisplatin, 5-fluorouracil; ECF: epirubicin, cisplatin, fluorouracil; ECOG: Eastern Cooperative Oncology Group; EOX: epirubicin, oxaliplatin, capecitabine; FLOT: fluorouracil, leucovorin, oxaliplatin, docetaxel; FOLFOX: folinic acid, fluorouracil and oxaliplatin; FOLFIRI: folinic acid, fluorouracil, irinotecan hydrochloride; 5FU: fluorouracil; GC: gastric cancer; GEJC: gastroesophageal junction cancer; PD-L1: programmed death-ligand 1; XELOX: capecitabine and oxaliplatin; XP: capecitabine and cisplatin.

There is a minimal role for surgery in incurable GC and GEJC beyond helping with supportive care procedures—for instance, placing a stent for obstruction or placing a gastric or jejunal feeding tube in those who require enteral feeding. However, there is the potential for conversion of unresectable into resectable disease following a good response to novel, targeted, or neoadjuvant therapy, although this is rare. Radiation therapy can be beneficial as a palliative therapy, especially in the setting of bleeding tumors or localized pain due to metastatic lesions. However, chemotherapy is the main treatment for unresectable metastatic GC and GEJC. With additional lines of therapy now available, such as trifluridine–tipiracil, these patients are able to continue receiving treatment into the third and fourth lines (Figure 1) [29]. The availability of multiple biomarkers and associated targeted therapies has improved first-line treatment decision-making. Tumors with overexpression of the HER2 biomarker are treated with anti-HER2 therapies, such as trastuzumab. MSI-H and PD-L1 biomarkers support the use of immunotherapy, although there is public funding for immunotherapy regardless of PD-L1 CPS results in Canada (Figure 1). New first-line data suggest improved outcomes for the addition of pembrolizumab to trastuzumab, fluoropyrimidine-, and platinum-containing chemotherapy for HER2-positive metastatic GC and GEJC with a PD-L1 CPS of ≥ 1 [30]. Furthermore, there have been positive results of phase III clinical trials reported for the addition of zolbetuximab to standard first-line chemotherapy for metastatic GC and GEJC for patients whose tumors overexpress CLDN18.2 [27,31]. With multiple first-line targeted therapies available, if combination data are not available, sequential therapy should be considered to avoid choosing between targeted therapies. Finally, while rare in metastatic GC and GEJC, for patients whose tumors are found to have a neurotrophic tropomyosin receptor kinase (NTRK) fusion, after progression on standard treatment, therapy with larotrectinib or entrectinib would be appropriate as per the NTRK-positive tumor agnostic indication [32,33]. There are multiple other emerging biomarkers for metastatic GC and GEJC treatment being explored, including mesenchymal-epithelial transition (MET) and fibroblast growth factor receptor 2 (FGFR2), which may provide new targets for personalized treatment [24]. As new biomarkers become available and targeted treatments are discovered, they should be included in the reflexive biomarker panel testing.

### 5.3. Clinical Trial Enrollment

Recommendation 3. Medical oncologists should offer all patients clinical trial enrollment if possible. Even with the best current clinical practice, outcomes for patients with GC and GEJC remain poor; best practices should include the consideration of enrollment in well-designed clinical trials [34]. However, there are challenges associated with enrolling patients in most clinical trials. Strict eligibility criteria driven by biomarker identification and performance status requirements can delay or restrict enrollment. The rapid progression of unresectable GC or GEJC disease can pose a serious risk, as the logistics of enrollment can take several weeks. Patients who are frail with a poor performance status are ineligible for clinical trials, while patients with preserved functional status and a large burden of disease or rapidly progressive symptoms are not able to wait while eligibility for a trial is assessed. It is particularly difficult to enroll patients in trials assessing first-line therapy, highlighting the value of biomarker testing at diagnosis to help facilitate timely trial enrollment and access to the best treatment. Furthermore, access to clinical trials may be difficult for patients living in centers without clinical trial units and may need to be referred to centers outside of their immediate geography.

### 5.4. Multidisciplinary Input on Treatment

Recommendation 4. All patients should be assessed at the time of diagnosis for relevant services and be cared for by collaborative and openly communicative providers. Multidisciplinary cancer conferences (MCCs) or tumor boards provide opportunities for a multidisciplinary review of challenging patient cases and the evaluation of potential treatment options to ensure optimal care. Retrospective studies have demonstrated that patient cases reviewed by high-volume multidisciplinary tumor boards have improved outcomes [35,36,37]. However, these formal meetings are not available in all centers. Medical management will focus on treatment decisions; allied health professionals will focus on specific patient support requirements (e.g., nutrition, family considerations, genetics, financial barriers, and social services). A multidisciplinary approach to caring for patients is critical. In centers that do not have access to all required resources, a multidisciplinary approach provides the opportunity for the care team to discuss key patient support considerations with opportunities for virtual and/or asynchronous multidisciplinary input from other well-resourced areas. It is important for oncologists to build relationships with allied health professionals and develop a strong network with other GC and GEJC specialists.

## 6. Nutritional Support and RD Collaboration

### 6.1. Value of RDs

Malnutrition in patients with GC and GEJC can have a significant impact on treatment tolerance, overall functioning, and QoL. Therefore, RDs are key players in optimizing a patient’s nutritional status to both address symptom management and introduce vitamin/minerals or nutrient preparations to ensure optimal physiologic functioning. It is important for an RD to be a primary contact for patient and caregiver questions related to appropriate foods and the use of over-the-counter supplements. In reality, the availability of dietitians, especially those with oncology expertise, varies by geographic location. Patients may need to be triaged based on the need to access dietitian services and support. Patients not requiring parenteral nutrition may be able to consult an RD virtually or by phone through RD telehealth services (available in some provinces, including British Columbia, Alberta, Saskatchewan, Manitoba, Ontario, Québec, and Nova Scotia). This type of service may be helpful for patients who may not need immediate supportive care measures and/or are interested in preventative care. Patients can also find dietitians through their Family Health Team practice or seek private practice dietitians through Dietitians of Canada. Better access to educational and peer-reviewed resources on optimal nutritional support for GC and GEJC patients is an unmet need. In some cases, patients may also seek naturopathic support to help manage nutritional concerns, but interventions could be contraindicated with cancer treatments.

### 6.2. Screening and RD Referral

Recommendation 5. Screening for malnutrition should be completed at the time of diagnosis and repeated throughout the patient’s journey, with early referral to an RD for those at nutritional risk. While most GC patients should ideally be referred to an RD upon diagnosis, current resource limitations in some jurisdictions have resulted in referrals based only on percentage weight loss. Priority is given to those who have lost >20% of their body weight, which implies reduced physiological capacity to consume or absorb an appropriate number of calories or nutrients. However, this criterion overlooks those who experience nutritional deficits without significant weight loss but with similar negative impacts on their health. Low muscle mass is strongly correlated with malnutrition and should be taken into consideration when evaluating the nutritional status of patients with GC and GEJC [38]. The presence of sarcopenia can lead to poor long-term prognoses for patients undergoing surgery for upper GC [14,15,16,17,38,39,40,41]. Therefore, gaining an understanding of patients’ dietary intake, factors affecting the ability to eat and/or absorb nutrients, reduced muscle mass, and muscle function are important considerations in addition to weight loss.

Patients with GC and GEJC should ideally undergo malnutrition screening at diagnosis and at regular intervals throughout treatment. Patients identified as being at risk for malnutrition should undergo a comprehensive nutrition assessment and intervention by an RD. Malnutrition as described by the Global Leadership Initiative on Malnutrition (GLIM) includes two sets of criteria: phenotypic (weight loss, low body mass index [BMI], and low skeletal muscle mass) and etiologic (low food intake and disease burden or inflammation) [42]. Many institutions do not have a standardized process for malnutrition screening despite the availability of validated malnutrition screening tools (Patient-generated Subjective Global Assessment [PG-SGA], Malnutrition Screening Tool [MST], Malnutrition Universal Screening Tool [MUST], Mini Nutritional Assessment [MNA], Nutrition Risk Screening [NRS2002], and NUTRISCORE). Barriers to using these tools include the time required to complete, lack of training, and limited resources to provide a meaningful intervention. Currently, the process for RD referrals is frequently reactive rather than proactive. Incorporating regular malnutrition screening into practice can lead to earlier diagnosis and treatment of malnutrition.

### 6.3. Peritoneal Involvement and Artificial Nutrition Support Using Enteral Nutrition and Parenteral Nutrition (EN/PN)

Recommendation 6. EN/PN should be considered for patients who struggle to consume at least 60% of their daily caloric intake for more than 7–14 days despite nutrition counseling, pharmacological interventions, and palliative care measures to enhance oral intake. EN/PN should be considered if it can improve systemic therapy outcomes and promote better symptom management. EN/PN should also be considered in patients who are severely malnourished as a result of uncontrolled disease to improve QoL, nutritional, and functional status [43,44]. Peritoneal involvement is often associated with malnutrition and an inability to tolerate EN, making these patients candidates for PN (EN is preferred to PN as it is less invasive and mimics the normal physiological process). Before initiating artificial nutritional support, it is important for the physician to talk with the patient about the goals, intended effects and timelines, and criteria for discontinuation. This support is often dependent on local availability, as each method may require setup in a hospital, which can impact treatment initiation timelines.

### 6.4. Nutrition Guidance for GC and GEJC Patients

Recommendation 7. Nutritional deficiencies in patients with GC and GEJC should ideally be corrected prior to treatment and palliative surgical procedures to improve patient outcomes in the perioperative setting. Malnutrition can cause negative outcomes in patients with cancer who are undergoing treatment, as demonstrated by the increased risk of poor treatment tolerance/delays, worsened symptoms, and worse overall survival [45]. Difficulty ingesting and assimilating adequate nutrition in unresectable GC due to anorexia–cachexia syndrome, caused mainly by the obstruction of the upper digestive tract due to mechanical effects of the tumor, and the use of previous treatments (i.e., prior chemotherapy, radiotherapy, and surgery) may contribute to compromised nutritional status [43]. Furthermore, previous surgery for GC may contribute to malnutrition, particularly micronutrient deficiencies. Current knowledge about the resulting micronutrient deficiencies is limited, with the exception of iron and vitamin B12. Guidance on how to recognize, treat, and prevent these deficiencies can be found in Table 2. Iron deficiency anemia (IDA) is a common occurrence with GC and GEJC due to ongoing tumor blood loss, in addition to being a complication following gastric surgery due to alterations in the digestion and impaired absorption of iron [43,46]. Thus, intravenous (IV) iron is a common supportive care measure given that most patients are iron deficient and/or have IDA at presentation and require rapid repletion [47]. Vitamin B12 requires gastric acid and intrinsic factor produced by parietal cells of the stomach for absorption, which is impaired by a gastrectomy. Although in some cases, vitamin B12 deficiency may not be detected for years due to a large hepatic storage, it is prevalent in patients before and early after gastrectomy. The potential coexistence of vitamin B12 and iron deficiency requires careful consideration when diagnosing post-gastrectomy anemia, as treatment may require the replenishment of both [48]. More research is needed to determine the incidence and management of other micronutrient deficiencies in this population. Since there are no nutritional guidelines for GC or GEJC, data and best practices from research on post-bariatric surgery micronutrient deficiencies and guidelines may provide insight into additional deficiencies that gastric cancer patients may be at risk of developing. Table 3 outlines these micronutrients and the signs/symptoms that may raise suspicion for a specific deficiency [49]. In all instances, issues such as impaired motility, malabsorption, and dumping syndrome should be considered and addressed when developing care plans to address micronutrient deficiencies.

## 7. Ongoing Role of Palliative Care

Recommendation 8. Palliative care should be integrated early to maximize supportive care. Unresectable GC/GEJC patients frequently have extensive supportive care needs due to the significant burden of their disease and adverse effects from therapy, including nausea, vomiting, fatigue, and pain. Early involvement of palliative care has demonstrated improvements in QoL and survival outcomes across many cancer types, which may allow patients to better tolerate anti-cancer therapies [62,63,64,65,66]. Survival in patients with unresectable GC/GEJC is modest in most cases, and the availability of palliative care teams can be highly variable across Canada, with potentially significant wait times; thus, early referral is recommended.

## 8. Adherence to Treatment and Supportive Care

Recommendation 9. Patient navigation of common barriers to treatment and supportive care requires a multidisciplinary care team. A patient’s ability to access and be adherent to treatment and supportive care is complex, involving physical, psychological, and financial factors. To help optimize treatment adherence, multidisciplinary care teams should be involved in symptom and adverse effect management, including the introduction of supportive medications (e.g., IV iron, anti-diarrheals, anti-emetics). Furthermore, patient follow-up should be regular and thorough to allow for prompt initiation of supportive medications or modification of their treatment plan. Ideally, patients should have access to a reputable resource to contact outside of scheduled follow-ups to address questions related to their treatment, such as the nursing call service provided by Ontario Health/Cancer Care Ontario (CCO).

The diagnosis of GC, especially at advanced and incurable stages, is frequently accompanied by emotional distress, which may impact a patient’s treatment adherence, QoL, functioning, pain, and, potentially, survival [67]. Patients may, therefore, benefit from a psychologist who focuses on navigating mental health challenges, including cancer-related fatigue, depression, and anxiety. Patients may also experience significant financial burdens due to the inability to work with increasing non-covered treatment-associated costs, such as travel or nutritional supplements. Social workers can help mitigate these issues by helping navigate the financial burden of treatment and ensuring that patients and their families are aware of resources and how to access them. Patients who have an established support system, including family and friends, social workers, and psychologists, demonstrate better treatment outcomes than those who do not or those who receive social work support only [68].

Anecdotally, patients are more likely to engage with supportive care measures, including RD services, psychosocial support, peer support groups (such as My Gut Feeling), and palliative care, if they experience the positive impact from them. For some patients, nausea, reduced appetite, and feelings of shame surrounding lapses in prescribed eating may impact their adherence to RDs’ recommendations. The efficacy of nutritional interventions may similarly be compromised if they are being asked to reduce the consumption of culture-specific foods, suggesting the need for culturally diverse resources for dietitians. Furthermore, the high cost of food, challenges with food preparation, and sanitary storage conditions may be barriers to some patients of low socioeconomic status. To encourage the success of dietary interventions, discussions about patients’ nutritional needs should involve the entire care team and a tailored approach.

In all supportive care, it is important to recognize any language barriers and utilize translated resources and/or interpretation services. If not appropriately addressed, language barriers can lead to a lack of knowledge, feelings of embarrassment, and avoidance of certain supportive care measures altogether.

## 9. Conclusions

GC and GEJC frequently progress quickly and are associated with malnutrition, both of which significantly impact a patient’s QoL and treatment tolerance. The need for earlier diagnosis, addressing comorbidities, nutritional support, and identification of relevant biomarkers with tailored treatment are crucial for overall survival. Where possible, patients should be offered access to appropriate clinical trials and explore effective treatment innovations. The need for a multidisciplinary approach with access to medical oncologists, surgeons, radiation oncologists, dietitians, palliative care teams, psychologists, and social workers is critical. Experts recommend collaborating with allied healthcare professionals to provide patients with the best possible outcomes while sustaining an adequate QoL.

## Figures and Tables

**Table 1:** Expert recommendations for the treatment and management of unresectable metastatic GC and GEJC.

Physician Recommendations
Impact of comorbidities on treatment (Section 4)
Physicians should determine the severity of comorbidities and their impacts on functional and performance status, and they should consider comorbidity management strategies prior to initiating treatment.
Diagnosis and treatment-specific considerations for patient management (Section 5.1)
2. Reflexive biomarker testing, including mismatch repair proteins (MMRs), programmed death-ligand 1 (PD-L1)/combined positive score (CPS), and human epidermal growth factor receptor 2 (HER2), should be standard practice at all centers to improve personalized care and outcomes.
Clinical Trial Enrollment (Section 5.3)
3. Recommendation 3. Medical oncologists should offer all patients clinical trial enrollment if possible.
Multidisciplinary input on treatment (Section 5.4)
4. All patients should be assessed at the time of diagnosis for relevant services and be cared for by collaborative and openly communicative providers.
Screening and dietitian referral (Section 6.2)
5. Screening for malnutrition should be completed at the time of diagnosis and repeated throughout the patient’s journey, with early referral to an RD for those at nutritional risk.
Peritoneal involvement and partial and/or total parenteral nutrition (PPN/TPN) (Section 6.3)
6. Enteral/parenteral nutrition (EN/PN) should be considered for patients who struggle to consume at least 60% of their daily caloric intake for more than 7–14 days despite nutrition counseling, pharmacological interventions, and palliative care measures to enhance oral intake.
Nutrition guidance for unresectable GC and GEJC patients (Section 6.4)
7. Recommendation 7. Nutritional deficiencies in patients with GC and GEJC should ideally be corrected prior to treatment and palliative surgical procedures to improve patient outcomes in the perioperative setting
Ongoing role of palliative care (Section 7)
8. Palliative care should be integrated early to maximize supportive care.
Social workers and psychotherapeutic support (Section 8)
9. Patient navigation of common barriers to treatment and supportive care requires a multidisciplinary care team.

**Table 2:** Guidelines on identifying and treating common micronutrient deficiencies in patients who have undergone surgery for gastric cancer.

Micronutrient	Signs/Symptoms of Deficiency	Supplementation
Iron [50]	Fatigue Shortness of breath Headache Pallor Anemia Pica or pagophagia Restless legs Angular cheilitis	IV iron (1000 mg) is preferred. If IV administration is not required, oral replacement of 150–300 mg 2–3 times per week should be considered if not contraindicated.
Vitamin B12 [51,52]	Anemia or pancytopenia Sensory predominant neuropathy Ataxia Glossitis Delirium Depression Skin hyper- or hypo-pigmentation	1000 mcg IM or SQ monthly is preferred. A nasal spray may also be considered.

IM: intramuscular; IV: intravenous; SQ: subcutaneous.

**Table 3:** Signs and symptoms that may be associated with micronutrient deficiencies in patients who have undergone surgery for GC.

Micronutrient	Symptom/Signs of Deficiency
Folate [53]	Anemia or pancytopenia Sensory predominant neuropathy Oral ulcers Neural tube defects in children of folate-deficient mothers
Vitamin A [54,55,56]	Vision/eye changes—initially night blindness—can progress to corneal damage or blindness Xerosis Bitot’s spots Hyperkeratinization of skin Loss of taste Poor healing
Vitamin D [52,55,56]	Paresthesia Cramping Tetany Muscle pain Demineralization of bones Hypocalcemia
Thiamine (Vitamin B1) [56,57]	Peripheral sensory neuropathy Ataxia Loss of reflexes Ataxia Encephalopathy Oculomotor dysfunction Impaired memory/learning Edema Vomiting
Zinc [52,58]	Impaired immune function Alopecia or change in hair color Sexual dysfunction Dysgeusia Night blindness Impaired wound healing Skin lesions
Copper [52,59]	Anemia and/or neutropenia Ataxia Neuropathy Fragile, abnormal hair texture Skin depigmentation Muscle weakness
Selenium [60]	Skeletal muscle dysfunction Cardiomyopathy Mood disorders Impaired immune function Whitened nailbeds Macrocytosis
Calcium [61]	Bone and/or tooth disease Secondary hyperparathyroidism Paresthesia Muscle Twitching Papilledema Laryngospasm/bronchospasm Arrhythmia

## References

[1] Sung H., Ferlay J., Siegel R.L., Laversanne M., Soerjomataram I., Jemal A., Bray F. (2021). Global Cancer Statistics 2020: GLOBOCAN Estimates of Incidence and Mortality Worldwide for 36 Cancers in 185 Countries. CA Cancer J. Clin..

[2] Brenner D.R., Poirier A., Woods R.R., Ellison L.F., Billette J.M., Demers A.A., Zhang S.X., Yao C., Finley C., Fitzgerald N. (2022). Projected estimates of cancer in Canada in 2022. CMAJ.

[3] Sekiguchi M., Oda I., Matsuda T., Saito Y. (2022). Epidemiological Trends and Future Perspectives of Gastric Cancer in Eastern Asia. Digestion.

[4] Bickenbach K., Strong V.E. (2012). Comparisons of Gastric Cancer Treatments: East vs. West. J. Gastric Cancer.

[5] Arnold M., Rutherford M.J., Bardot A., Ferlay J., Andersson T.M., Myklebust T., Tervonen H., Thursfield V., Ransom D., Shack L. (2019). Progress in cancer survival, mortality, and incidence in seven high-income countries 1995–2014 (ICBP SURVMARK-2): A population-based study. Lancet Oncol..

[6] Oh J., Abboud Y., Burch M., Gong J., Waters K., Ghaith J., Jiang Y., Park K., Liu Q., Watson R. (2023). Rising Incidence of Non-Cardia Gastric Cancer among Young Women in the United States, 2000–2018: A Time-Trend Analysis Using the USCS Database. Cancers.

[7] Morgan E., Arnold M., Camargo M.C., Gini A., Kunzmann A.T., Matsuda T., Meheus F., Verhoeven R.H.A., Vignat J., Laversanne M. (2022). The current and future incidence and mortality of gastric cancer in 185 countries, 2020–2040: A population-based modelling study. EClinicalMedicine.

[8] Reyes V.E. (2023). Helicobacter pylori and its role in gastric cancer. Microorganisms.

[9] Zhang Y., Zhang P.S., Rong Z.Y., Huang C. (2021). One stomach, two subtypes of carcinoma-the differences between distal and proximal gastric cancer. Gastroenterol. Rep..

[10] Mukkamalla S.R., Recio-Boiles A., Babiker H.M. (2023). Gastric Cancer. StatPearls.

[11] Alghamdi A.G., Alshareef A.M., Alzahrani A.T., Alharthi Z.S., Alghamdi S.S., Alghamdi A.M., Alzahrani F.A., Alzahrani R.A. (2023). Knowledge and Awareness About Gastric Cancer Among the General Population in Al-Baha City, Saudi Arabia. Cureus.

[12] Lim H.S., Lee B., Cho I., Cho G.S. (2020). Nutritional and Clinical Factors Affecting Weight and Fat-Free Mass Loss after Gastrectomy in Patients with Gastric Cancer. Nutrients.

[13] Deane A.M., Chapman M.J., Reintam Blaser A., McClave S.A., Emmanuel A. (2019). Pathophysiology and Treatment of Gastrointestinal Motility Disorders in the Acutely Ill. Nutr. Clin. Pract..

[14] Cai W., Yang H., Zheng J., Huang J., Ji W., Lu Y., Yang X., Zhang W., Shen X., Chen X. (2022). Global leaders malnutrition initiative-defined malnutrition affects long-term survival of different subgroups of patients with gastric cancer: A propensity score-matched analysis. Front. Nutr..

[15] Karabulut S., Dogan I., Usul Afsar C., Karabulut M., Ak N., Duran A., Tastekin D. (2022). Does nutritional status affect treatment tolerability, chemotherapy response and survival in metastatic gastric cancer patients? Results of a prospective multicenter study in Turkey. J. Oncol. Pharm. Pract..

[16] Marshall K.M., Loeliger J., Nolte L., Kelaart A., Kiss N.K. (2019). Prevalence of malnutrition and impact on clinical outcomes in cancer services: A comparison of two time points. Clin. Nutr..

[17] Gharagozlian S., Mala T., Brekke H.K., Kolbjornsen L.C., Ullerud A.A., Johnson E. (2020). Nutritional status, sarcopenia, gastrointestinal symptoms and quality of life after gastrectomy for cancer—A cross-sectional pilot study. Clin. Nutr. ESPEN.

[18] Tanaka C., Kanda M., Murotani K., Yoshikawa T., Cho H., Ito Y., Matsui T., Nakayama H., Yamada T., Kobayashi D. (2019). Long-term quality of life and nutrition status of the aboral pouch reconstruction after total gastrectomy for gastric cancer: A prospective multicenter observational study (CCOG1505). Gastric Cancer.

[19] GlobalSurg C., Surgery N.G.H.U.o.G. (2023). Impact of malnutrition on early outcomes after cancer surgery: An international, multicentre, prospective cohort study. Lancet Glob. Health.

[20] Katiyar V., Vohra I., Gopakumar H., Sharma V.R. (2023). Impact of malnutrition on postoperative outcomes of patients undergoing gastrectomy for gastric cancer: A nationwide analysis between 2012 and 2017. J Clin Oncol..

[21] Isobe T., Hashimoto K., Kizaki J., Miyagi M., Aoyagi K., Koufuji K., Shirouzu K. (2013). Characteristics and prognosis of gastric cancer in young patients. Oncol. Rep..

[22] Loizides S., Papamichael D. (2022). Considerations and Challenges in the Management of the Older Patients with Gastric Cancer. Cancers.

[23] Necula L., Matei L., Dragu D., Neagu A.I., Mambet C., Nedeianu S., Bleotu C., Diaconu C.C., Chivu-Economescu M. (2019). Recent advances in gastric cancer early diagnosis. World J. Gastroenterol..

[24] Choi S., Park S., Kim H., Kang S.Y., Ahn S., Kim K.M. (2022). Gastric Cancer: Mechanisms, Biomarkers, and Therapeutic Approaches. Biomedicines.

[25] Janjigian Y.Y., Shitara K., Moehler M., Garrido M., Salman P., Shen L., Wyrwicz L., Yamaguchi K., Skoczylas T., Campos Bragagnoli A. (2021). First-line nivolumab plus chemotherapy versus chemotherapy alone for advanced gastric, gastro-oesophageal junction, and oesophageal adenocarcinoma (CheckMate 649): A randomised, open-label, phase 3 trial. Lancet.

[26] Amonkar M., Lorenzi M., Zhang J., Mehta S., Liaw K.-L. (2019). Structured literature review (SLR) and meta-analyses of the prevalence of microsatellite instability high (MSI-H) and deficient mismatch repair (dMMR) in gastric, colorectal, and esophageal cancers. J. Clin. Oncol..

[27] Shitara K., Lordick F., Bang Y.J., Enzinger P., Ilson D., Shah M.A., Van Cutsem E., Xu R.H., Aprile G., Xu J. (2023). Zolbetuximab plus mFOLFOX6 in patients with CLDN18.2-positive, HER2-negative, untreated, locally advanced unresectable or metastatic gastric or gastro-oesophageal junction adenocarcinoma (SPOTLIGHT): A multicentre, randomised, double-blind, phase 3 trial. Lancet.

[28] Lian J., Zhang G., Zhang Y., Liu H., Zhang J., Nan P., Tian W. (2022). PD-L1 and HER2 expression in gastric adenocarcinoma and their prognostic significance. Dig. Liver Dis..

[29] Shitara K., Doi T., Dvorkin M., Mansoor W., Arkenau H.T., Prokharau A., Alsina M., Ghidini M., Faustino C., Gorbunova V. (2018). Trifluridine/tipiracil versus placebo in patients with heavily pretreated metastatic gastric cancer (TAGS): A randomised, double-blind, placebo-controlled, phase 3 trial. Lancet Oncol..

[30] Janjigian Y.Y., Kawazoe A., Bai Y., Xu J., Lonardi S., Metges J.P., Yanez P., Wyrwicz L.S., Shen L., Ostapenko Y. (2023). Pembrolizumab plus trastuzumab and chemotherapy for HER2-positive gastric or gastro-oesophageal junction adenocarcinoma: Interim analyses from the phase 3 KEYNOTE-811 randomised placebo-controlled trial. Lancet.

[31] Xu R.-H., Shitara K., Ajani J.A., Bang Y.-J., Enzinger P.C., Ilson D.H., Lordick F., Van Cutsem E., Gallego Plazas J., Huang J. (2023). Zolbetuximab + CAPOX in 1L claudin-18.2+ (CLDN18.2+)/HER2− locally advanced (LA) or metastatic gastric or gastroesophageal junction (mG/GEJ) adenocarcinoma: Primary phase 3 results from GLOW. J. Clin. Oncol..

[32] Doebele R.C., Drilon A., Paz-Ares L., Siena S., Shaw A.T., Farago A.F., Blakely C.M., Seto T., Cho B.C., Tosi D. (2020). Entrectinib in patients with advanced or metastatic NTRK fusion-positive solid tumours: Integrated analysis of three phase 1–2 trials. Lancet Oncol..

[33] Shen L., Andre T., Chung H.C.C., Deeken J.F., Garralda E., Italiano A., Leyvraz S., Liu T., Burcoveanu D.-I., Grugel R. (2024). Updated efficacy and safety of larotrectinib (laro) in patients (pts) with TRK fusion gastrointestinal (GI) cancer. J. Clin. Oncol..

[34] Ajani J.A., D’Amico T.A., Bentrem D.J., Chao J., Cooke D., Corvera C., Das P., Enzinger P.C., Enzler T., Fanta P. (2022). Gastric Cancer, Version 2.2022, NCCN Clinical Practice Guidelines in Oncology. J. Natl. Compr. Cancer Netw..

[35] Creighton N., Walton R., Roder D.M., Aranda S., Richardson A.J., Merrett N., Currow D. (2017). Pancreatectomy is underused in NSW regions with low institutional surgical volumes: A population data linkage study. Med. J. Aust..

[36] Burmeister E.A., O’Connell D.L., Jordan S.J., Goldstein D., Merrett N., Wyld D.K., Beesley V.L., Gooden H.M., Janda M., Neale R.E. (2016). Factors associated with quality of care for patients with pancreatic cancer in Australia. Med. J. Aust..

[37] Smith R.C., Creighton N., Lord R.V., Merrett N.D., Keogh G.W., Liauw W.S., Currow D.C. (2014). Survival, mortality and morbidity outcomes after oesophagogastric cancer surgery in New South Wales, 2001–2008. Med. J. Aust..

[38] Lidoriki I., Schizas D., Mpaili E., Vailas M., Sotiropoulou M., Papalampros A., Misiakos E., Karavokyros I., Pikoulis E., Liakakos T. (2019). Associations between skeletal muscle mass index, nutritional and functional status of patients with oesophago-gastric cancer. Clin. Nutr. ESPEN.

[39] Liu T., Yi X., Ge J., Zhang J., Tan F., Song K., Liu H., Tang M. (2022). Preoperative computed tomography-determined sarcopenia is a reliable prognostic factor in patients with gastric cancer after radical gastrectomy: A sex-specific analysis. Front. Nutr..

[40] Park A., Orlandini M.F., Szor D.J., Junior U.R., Tustumi F. (2023). The impact of sarcopenia on esophagectomy for cancer: A systematic review and meta-analysis. BMC Surg..

[41] Juez L.D., Priego P., Bajawi M., Cuadrado M., Blázquez L.A., Sánchez-Picot S., Galindo J., Blázquez J., Fernández-Cebrián J.M., Botella-Carretero J.I. (2023). Impact of Sarcopenic Obesity on Long-Term Cancer Outcomes and Postoperative Complications After Gastrectomy for Gastric Cancer. J. Gastrointest. Surg..

[42] Barazzoni R., Jensen G.L., Correia M., Gonzalez M.C., Higashiguchi T., Shi H.P., Bischoff S.C., Boirie Y., Carrasco F., Cruz-Jentoft A. (2022). Guidance for assessment of the muscle mass phenotypic criterion for the Global Leadership Initiative on Malnutrition (GLIM) diagnosis of malnutrition. Clin. Nutr..

[43] Rosania R., Chiapponi C., Malfertheiner P., Venerito M. (2016). Nutrition in Patients with Gastric Cancer: An Update. Gastrointest. Tumors.

[44] Zhang Y., Zhang J., Zhu L., Hao J., He F., Xu T., Wang R., Zhuang W., Wang M. (2023). A Narrative Review of Nutritional Therapy for Gastrointestinal Cancer Patients Underwent Surgery. J. Investig. Surg..

[45] Matsui R., Rifu K., Watanabe J., Inaki N., Fukunaga T. (2023). Impact of malnutrition as defined by the GLIM criteria on treatment outcomes in patients with cancer: A systematic review and meta-analysis. Clin. Nutr..

[46] Tang G.H., Hart R., Sholzberg M., Brezden-Masley C. (2018). Iron deficiency anemia in gastric cancer: A Canadian retrospective review. Eur. J. Gastroenterol. Hepatol..

[47] Rostamnjad L., Leblebjian H., Falb J., Dolan M., Sommer K.A., McCleary N.J. (2022). Evaluation of clinical use of intravenous iron: Utilization, efficacy, and safety in the management of cancer and chemotherapy-induced anemia in GI oncology. J. Clin. Oncol..

[48] Ao M., Awane M., Asao Y., Kita S., Miyawaki T., Tanaka K. (2023). High prevalence of vitamin B-12 deficiency before and early after gastrectomy in patients with gastric cancer. Asia Pac. J. Clin. Nutr..

[49] Lupoli R., Lembo E., Saldalamacchia G., Avola C.K., Angrisani L., Capaldo B. (2017). Bariatric surgery and long-term nutritional issues. World J. Diabetes.

[50] Obinwanne K.M., Fredrickson K.A., Mathiason M.A., Kallies K.J., Farnen J.P., Kothari S.N. (2014). Incidence, treatment, and outcomes of iron deficiency after laparoscopic Roux-en-Y gastric bypass: A 10-year analysis. J. Am. Coll. Surg..

[51] Pech N., Meyer F., Lippert H., Manger T., Stroh C. (2012). Complications and nutrient deficiencies two years after sleeve gastrectomy. BMC Surg..

[52] Mechanick J.I., Apovian C., Brethauer S., Garvey W.T., Joffe A.M., Kim J., Kushner R.F., Lindquist R., Pessah-Pollack R., Seger J. (2019). Clinical practice guidelines for the perioperative nutrition, metabolic, and nonsurgical support of patients undergoing bariatric procedures—2019 update: Cosponsored by American Association of Clinical Endocrinologists/American College of Endocrinology, The Obesity Society, American Society for Metabolic & Bariatric Surgery, Obesity Medicine Association, and American Society of Anesthesiologists. Endocr. Pract..

[53] Brolin R.E., Gorman R.C., Milgrim L.M., Kenler H.A. (1991). Multivitamin prophylaxis in prevention of post-gastric bypass vitamin and mineral deficiencies. Int. J. Obes..

[54] Zalesin K.C., Miller W.M., Franklin B., Mudugal D., Rao Buragadda A., Boura J., Nori-Janosz K., Chengelis D.L., Krause K.R., McCullough P.A. (2011). Vitamin a deficiency after gastric bypass surgery: An underreported postoperative complication. J. Obes..

[55] Aasheim E.T., Björkman S., Søvik T.T., Engström M., Hanvold S.E., Mala T., Olbers T., Bøhmer T. (2009). Vitamin status after bariatric surgery: A randomized study of gastric bypass and duodenal switch. Am. J. Clin. Nutr..

[56] Peterson L.A., Cheskin L.J., Furtado M., Papas K., Schweitzer M.A., Magnuson T.H., Steele K.E. (2016). Malnutrition in Bariatric Surgery Candidates: Multiple Micronutrient Deficiencies Prior to Surgery. Obes. Surg..

[57] Lakhani S.V., Shah H.N., Alexander K., Finelli F.C., Kirkpatrick J.R., Koch T.R. (2008). Small intestinal bacterial overgrowth and thiamine deficiency after Roux-en-Y gastric bypass surgery in obese patients. Nutr. Res..

[58] Sallé A., Demarsy D., Poirier A.L., Lelièvre B., Topart P., Guilloteau G., Bécouarn G., Rohmer V. (2010). Zinc deficiency: A frequent and underestimated complication after bariatric surgery. Obes. Surg..

[59] Gletsu-Miller N., Broderius M., Frediani J.K., Zhao V.M., Griffith D.P., Davis S.S., Sweeney J.F., Lin E., Prohaska J.R., Ziegler T.R. (2012). Incidence and prevalence of copper deficiency following roux-en-y gastric bypass surgery. Int. J. Obes..

[60] Shahmiri S.S., Eghbali F., Ismaeil A., Gholizadeh B., Khalooeifard R., Valizadeh R., Rokhgireh S., Kermansaravi M. (2022). Selenium Deficiency After Bariatric Surgery, Incidence and Symptoms: A Systematic Review and Meta-Analysis. Obes. Surg..

[61] Shah M., Sharma A., Wermers R.A., Kennel K.A., Kellogg T.A., Mundi M.S. (2017). Hypocalcemia After Bariatric Surgery: Prevalence and Associated Risk Factors. Obes. Surg..

[62] Gautama M.S.N., Damayanti A., Khusnia A.F. (2023). Impact of Early Palliative Care to Improve Quality of Life of Advanced Cancer Patients: A Meta-Analysis of Randomised Controlled Trials. Indian. J. Palliat. Care.

[63] Kim C., Lelond S., Daeninck P.J., Rabbani R., Lix L., McClement S., Chochinov H., Goldenberg B.A. (2021). The impact of early palliative care on the quality of life of patients with advanced pancreatic cancer: The IMPERATIVE study. J. Clin. Oncol..

[64] Seow H., Sutradhar R., Burge F., McGrail K., Guthrie D.M., Lawson B., Oz U.E., Chan K., Peacock S., Barbera L. (2021). End-of-life outcomes with or without early palliative care: A propensity score matched, population-based cancer cohort study. BMJ Open.

[65] Shih H.H., Chang H.J., Huang T.W. (2022). Effects of Early Palliative Care in Advanced Cancer Patients: A Meta-Analysis. Am. J. Hosp. Palliat. Care.

[66] Harada K., Zhao M., Shanbhag N., Baba H., Ajani J.A. (2020). Palliative care for advanced gastric cancer. Expert. Rev. Anticancer Ther..

[67] Kim G.M., Kim S.J., Song S.K., Kim H.R., Kang B.D., Noh S.H., Chung H.C., Kim K.R., Rha S.Y. (2017). Prevalence and prognostic implications of psychological distress in patients with gastric cancer. BMC Cancer.

[68] Bou-Samra P., Scott P., Cheng H., Kallem C., Pathak R., Geller D.A., Marsh W., Wang Y., Antoni M., Penedo F. (2022). Social Support is Associated with Survival in Patients Diagnosed with Gastrointestinal Cancer. J. Gastrointest. Cancer.

